# KillerOrange, a Genetically Encoded Photosensitizer Activated by Blue and Green Light

**DOI:** 10.1371/journal.pone.0145287

**Published:** 2015-12-17

**Authors:** Karen S. Sarkisyan, Olga A. Zlobovskaya, Dmitry A. Gorbachev, Nina G. Bozhanova, George V. Sharonov, Dmitriy B. Staroverov, Evgeny S. Egorov, Anastasia V. Ryabova, Kyril M. Solntsev, Alexander S. Mishin, Konstantin A. Lukyanov

**Affiliations:** 1 Shemyakin-Ovchinnikov Institute of Bioorganic Chemistry, Moscow, Russia; 2 Faculty of Biology, Moscow State University, Moscow, Russia; 3 Faculty of Medicine, Moscow State University, Moscow, Russia; 4 Pirogov Russian National Research Medical University, Moscow, Russia; 5 Laser Biospectroscopy Laboratory, Prokhorov General Physics Institute, Moscow, Russia; 6 School of Chemistry and Biochemistry, Georgia Institute of Technology, Atlanta, Georgia, 30332, United States of America; 7 Nizhny Novgorod State Medical Academy, Nizhny Novgorod, Russia; Cardiff University, UNITED KINGDOM

## Abstract

Genetically encoded photosensitizers, proteins that produce reactive oxygen species when illuminated with visible light, are increasingly used as optogenetic tools. Their applications range from ablation of specific cell populations to precise optical inactivation of cellular proteins. Here, we report an orange mutant of red fluorescent protein KillerRed that becomes toxic when illuminated with blue or green light. This new protein, KillerOrange, carries a tryptophan-based chromophore that is novel for photosensitizers. We show that KillerOrange can be used simultaneously and independently from KillerRed in both bacterial and mammalian cells offering chromatic orthogonality for light-activated toxicity.

## Introduction

Fluorescent proteins of GFP family are widely used in molecular and cellular biology as passive reporters of the cellular structures and processes [1]. The chromophore of a fluorescent protein is buried deeply inside a protein beta-barrel and shielded from the surrounding solvent. This is believed to explain a much lower level of phototoxicity of a typical fluorescent protein comparing to small organic dyes [2,3].

In one documented case, however, the structure of beta-barrel maintains a path through the protein that reaches the chromophore and is accessible by the water and oxygen molecules [4,5]. This unusual example belongs to the red fluorescent protein KillerRed developed from the jellyfish chromoprotein anm2CP by directed evolution [6]. When illuminated with green or orange light, KillerRed produces reactive oxygen species and demonstrates three orders of magnitude higher level of phototoxicity than other fluorescent proteins [6].

It was shown that the reactive oxygen species formation in KillerRed proceeds through the Type I (radical-based), and not the Type II (singlet oxygen formation) mechanism [7]. The mechanism of KillerRed action possibly involves unusual reductive electron transfer to the singlet excited protein chromophore forming the radical dianion intermediate [8]. The latter subsequently transfers an electron to the oxygen molecule forming the superoxide anion radical.

Depending on the subcellular localization and the dose of light irradiation, reactive oxygen species produced by KillerRed can lead to a variety of outcomes ranging from inactivation of a fusion partner protein [6], DNA damage and cell division arrest [9,10] to cell death through necrosis or apoptosis [6,11].

KillerRed is thus used as an optogenetic tool in cell biology to inactivate particular proteins by light [6,12–14] and to study local intracellular oxidative stress [15,16], and in developmental biology and neuroscience to ablate a specific cell population [17–21]. Besides that, KillerRed has been used as a photosensitizer for tumor treatment in model systems [22–25]

KillerRed was the first genetically encoded photosensitizer developed in 2006 [6]. Since then a few other proteins that generate reactive oxygen species have been reported: SuperNova [26], the spectrally similar monomeric version of KillerRed, and miniSOG and Pp2FbFP L30M–flavoproteins that generate singlet oxygen upon *blue* light irradiation mainly via Type II mechanism [27,28]. Together they cover the spectral range from 430 to 590 nm that can be used for reactive oxygen species production.

In the current work we aimed to further expand the toolkit of available phototoxic proteins by developing a KillerRed mutant of distinct color. Our goal was to develop a protein that can be excited independently of KillerRed, so the concurrent biomolecular processes or cell populations fates can be regulated in an orthogonal and non-interfering fashion. Such fine tuning leading to high selectivity is rapidly developing in a number of chemical and biochemical systems [29,30].

## Methods

### Plasmid construction and mutagenesis

Synthetic DNA oligonucleotides for mutagenesis were purchased from Evrogen. PCRs were carried out using PTC-100 thermal cycler (MJ Research). Purification of PCR products and products of digestion was performed by gel electrophoresis and extraction using the Cleanup Standard Kit (Evrogen). Site-directed mutagenesis was performed by self-assembly cloning [31].

Random mutagenesis was performed by error-prone PCR [32] using an in house analogue of the Diversify PCR Random Mutagenesis kit (Clontech, 50x mix: 0.2 мМ dGTP, 0.2 мМ dATP, 1 мМ dCTP, 1 мМ dTTP). The average mutation rate, which is a function of buffer composition and number of PCR cycles, was selected to result in approximately 2–3 amino acid mutations per gene sequence.

For bacterial expression, a fluorescent protein gene was cloned into the pQE-30 vector (Qiagen) using EcoRI and HindIII restriction sites (in random mutagenesis experiments) or by self-assembly cloning [33]. Restriction endonucleases were purchased from Sibenzyme. We selected mutants by visual screening of *Escherichia coli* colonies expressing mutant proteins using Olympus SZX12 fluorescence stereo zoom microscope.

The plasmid for the expression of mitochondria-targeted KillerOrange was constructed by inserting KillerOrange coding sequence into the pKillerRed-dMito vector (Evrogen) instead of KillerRed sequence (using AgeI and NotI restriction sites).

### Purification and characterization proteins in vitro

Fluorescent proteins were expressed in *E*. *coli* XL1 Blue strain (Invitrogen) in the LB medium, centrifuged, sonicated in PBS (pH 7.4), then purified using Talon metal-affinity resin (Clontech). Cary 100 UV/VIS Spectrophotometer and Varian Cary Eclipse Fluorescence spectrophotometer were used to measure absorption and excitation-emission spectra. Quantum yield was determined by direct comparison with TagRFP [34].

Extinction coefficient was determined as follows. Synthetic CFP chromophore in basic dimethylformamide possesses double-peaked absorption curve with two close maxima at 460 and 473 nm and extinction coefficient 46000 M^−1^cm^−1^ at both wavelengths [35]. This value was used to calculate the concentration of mature protein in a sample of alkali-denatured KillerOrange (these conditions are believed to result in the reduction of acylimin bond leading to the formation of CFP chromophore from KillerOrange’s mature chromophore). The extinction coefficients of the protein were then obtained spectrophotometrically using the known concentration.

To measure the pH dependence of spectra, we used buffers in the pH range from 3 to 11 (citrate buffers in the pH range 3.0–4.5, phosphate buffers in the pH range 5.0–7.5, and borate buffers in the pH range 8.0–11.0). An aliquot of purified protein was diluted in the corresponding buffer solution. Spectra were measured after about 15 min of incubation in buffer.

### Phototoxicity test in *E*. *coli*

To compare the phototoxicity in bacteria, we mixed a suspension of *E*. *coli* XL1 Blue cells (PBS pH 7.4, PanEco) transformed with EGFP (non-phototoxic control) with the suspension of cells transformed with the phototoxic protein. The *E*. *coli* cells used for the experiment were incubated overnight at 4°C to increase the fraction of the mature protein. We used genes cloned in pQE-30 vector under the leaky promoter and did not use induction for expression.

We diluted suspension to the optical density of 0.05 and aliquoted it into transparent PCR tubes, 40 mkl of the suspension per tube. We kept one aliquot in the darkness while others were subjected to illumination with light. All aliquots were then spread over the Petri dishes, cultivated for 12–16 hours at 37°C, washed out and analyzed with fluorescence-activated cell sorter (FACS Aria III, BD Biosciences).

For the experiment with EGFP-KillerOrange mixture we illuminated the sample for 60 seconds with custom LED illuminator (assembly of 7 Luxeon Star LXML-PB01-0040 Rebel LEDs, Lumileds, 470/20 nm, spectral half-width of 20 nm), with the light intensity of about 1 W/cm^2^ at the sample.

For the experiment with EGFP-KillerOrange-KillerRed mixture we illuminated samples with one of LEDs of X-Cite XLED1 illuminator (Lumen Dynamics Group Inc.) We used LEDs named “BDX” (450–495 nm), “BGX” (505–545 nm) and “GYX” (540–600 nm), their full spectra are shown at S3 Fig. The power of all LEDs was set to be about 80 mW/cm^2^ at the sample.

### Phototoxicity test in mammalian cells

The phototoxicity test in mammalian cells followed the same principle as the test in bacteria. We grew HEK 293 cells in DMEM (Paneco, Russia) on 6-cm Petri dishes until about 70% confluency. We then transiently transfected them using FuGene HD (Roche) with one of the following plasmids: pKillerRed-dMito (Evrogen), pKillerOrange-dMito (for this construct we replaced KillerRed coding sequence with KillerOrange in pKillerRed-dMito) or pEGFP-N1 (Clontech). We then imaged cells using a Zeiss LSM-710 confocal inverted microscope (Carl Zeiss, Germany) to confirm correct protein localization.

24 hours after transfection we washed cells off from the dish, centrifuged, resuspended in PBS pH7.4 (Paneco, Russia) and mixed in pairs (KillerOrange with EGFP, KillerRed with EGFP) in polystyrene optical cuvettes (Sarstedt, Germany). We then splitted suspensions in two halves, illuminated first one with custom LEDs while keeping the second in the darkness for the same amount of time. For blue light illumination we used assembly of 7 Luxeon Star LXML-PR01-0500 Rebel LEDs, Lumileds (dominant wavelength is 447 nm, spectral half-width is 20 nm), for orange illumination we used 7 Luxeon Star 7 LXML-PL01-0040 Rebel LEDs, Lumileds (dominant wavelength is 590 nm, spectral half-width is 20 nm). The light power at the sample was set to be about 60 mW for 447 nm light and about 20 mW for 590 nm light.

After illumination we transferred the cells back to Petri dishes, incubated cells for 24 hours, washed off again, and analyzed cell fluorescence with flow cytometer (Beckman Coulter, USA).

### TCSPC measurements

We measured fluorescence lifetime using an Edinburgh Instruments time-correlated single photon counting (TCSPC) system. Fluorescence was excited with a 467 nm picosecond excitation pulses diode laser (10 MHz, Picoquant) and measured by a detection system consisted of a high speed MicroChannel Plate PhotoMultiplier Tube (MCP-PMT, Hamamatsu R3809U-50) and TCSPC electronics. The time resolution of the system was 30 ps after deconvolution with an IRF signal.

## Results

**Fig 1:**
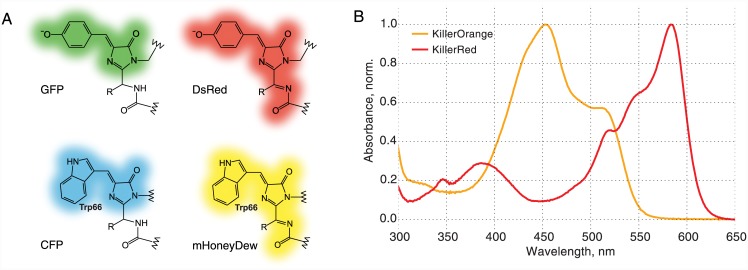
Structures of discussed chromophore types, absorption spectra of KillerRed and KillerOrange. **(A)** Structures of GFP, CFP, DsRed and mHoneyDew chromophores. **(B)** Normalized absorption spectra of KillerRed and KillerOrange.

KillerRed possesses a DsRed-like chromophore (Fig 1a) [4,5]. Proteins carrying this type of chromophore were shown to pass through the green- or blue-emitting states before their chromophore matures into the red-emitting structure [2]. In order to develop a non-red mutant we first performed a round of random mutagenesis on KillerRed gene expecting to find a substitution that disrupts acylimine group formation during the chromophore maturation. However, visual screening of about 100,000 colonies did not yield any green- or blue-fluorescent mutants.

We thus changed the strategy and made three mutants of KillerRed with substitutions of the chromophore’s tyrosine (Tyr66) by other aromatic amino acids. Y66H and Y66F substitutions made KillerRed completely non-fluorescent, and we failed to find compensatory substitutions during the following round of random mutagenesis. The mutant with Y66W substitution was weakly fluorescent in orange, and we managed to enhance its brightness in three rounds of random mutagenesis. The final mutant carrying 7 substitutions (KillerRed G3C Y66W D113S N145S F177L Y221H E236Q, see the full protein sequence as S1 Text) was a bright orange fluorescent protein with excitation maximum at 512 nm and emission maximum at 555 nm. We named it KillerOrange.

**Fig 2:**
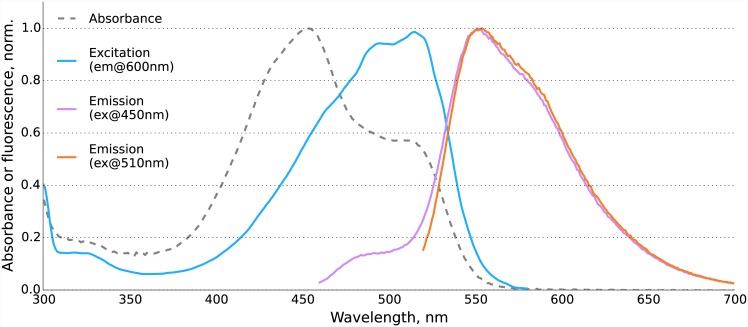
Absorption, excitation and emission spectra of KillerOrange.

Absorbance spectrum of KillerOrange demonstrated two peaks at 455 and 514 nm (Figs 1b and 2) likely corresponding to an immature CFP-like chromophore and a mature mHoneyDew-like tryptophan-based acylimine-containing chromophore [36], respectively (Fig 1a). Both forms seemed to possess the double-peak signature characteristic for neutral tryptophan-based chromophores [37]. We noticed that the ratio of the two peaks was influenced by the expression conditions, for example, expression on Petri dishes could result in a more complete maturation of the chromophore as compared with the expression in a liquid medium. The extinction coefficients of KillerOrange expressed in liquid medium were 41200 M^-1^·cm^-1^ at 455 nm and 22600 M^-1^·cm^-1^ at 514 nm. Despite the presence of two forms, the emission of the protein was almost purely orange (Fig 2) with the maximum at 555 nm (Stokes shift of over 40 nm) and quantum yield of 0.42. The immature form contributes little to the emitted light despite being dominant on the absorption spectrum due to its low quantum yield and/or efficient Förster resonance energy transfer within a dimer.

The fluorescence decay of KillerOrange was polyexponential, with the average fluorescence lifetime of 1.5 ns at pH 5.5, 1.9 ns at pH 7.4, and 2.6 ns at pH 9.5 (S1 Fig). The increase in fluorescence lifetime at alkaline conditions was accompanied by an increase in fluorescence intensity, as can be seen from the pH-dependence of KillerOrange spectra (S2 Fig), indicating the rise of quantum yield over pH.

To assess the phototoxicity of KillerOrange under various light conditions we designed an assay for *Escherichia coli* based on fluorescence-activated cell sorting. Briefly, we mixed *E*. *coli* cells expressing EGFP with *E*. *coli* cells expressing KillerOrange, and illuminated half of the suspension, while keeping another half in darkness. We then plated each aliquot on Petri dishes, grew overnight, washed the bacteria off from the plates, and analyzed the KillerOrange/EGFP ratio in the suspension using fluorescence-activated cell sorting. This approach assays only the *relative* phototoxicity of a protein, but is more precise and less time-consuming than the approach used earlier [38].

**Fig 3:**
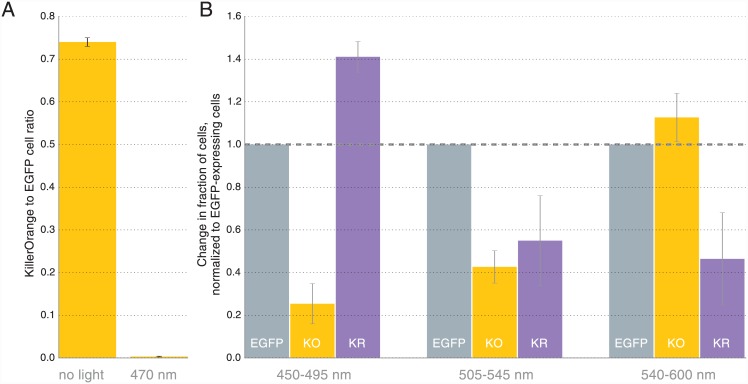
Phototoxicity of KillerOrange and KillerRed in *E*. *coli*. (**A**) Blue light (470/20) nm illumination results in selective elimination (222-fold) of KillerOrange-expressing cells from the mixture with EGFP-expressing cells. Error bars represent SD, N = 3. (**B**) KillerOrange (KO) and KillerRed (KR) toxicity under light illumination at different wavelengths. Error bars represent SD, N = 4.

Using a very intense blue light (470/20 nm, 1 W/cm^2^) we found that KillerOrange-expressing cells almost disappeared (222-fold decrease) from the population as a result of just 60 seconds of illumination (Fig 3a) confirming the high level of phototoxicity of KillerOrange.

To test if KillerOrange and KillerRed can be used independently to kill selected cell populations with light of different wavelengths we have performed similar experiment with three proteins. We mixed *E*. *coli* cells expressing KillerOrange with the cells expressing EGFP and cells expressing KillerRed. Using another light source and milder intensities, we illuminated aliquots of cell suspension with 450–495, 505–545 and 540–600 nm light (LED spectra are shown in S3 Fig). Illumination with 540–600 nm light resulted in the selective removal of KillerRed-expressing cells, while the illumination with 450–495 nm light killed the majority of KillerOrange-expressing cells (Fig 3b, S4 Fig). Interestingly, 505–545 nm light illumination was almost equally efficient in killing both KillerOrange and KillerRed cells. Thus, by combining different light sources one can achieve precise control over cell populations expressing KillerOrange and KillerRed.

**Fig 4:**
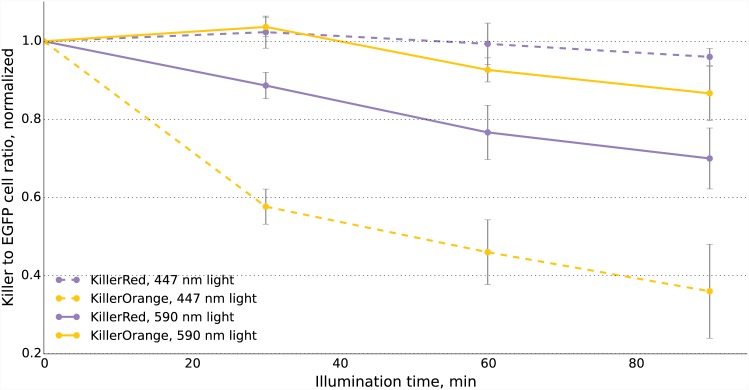
Phototoxicity of KillerOrange and KillerRed in mammalian cells. KillerOrange, KillerRed and EGFP-expressing cells were mixed and illuminated with 477 nm or 590 nm light. Y-axis depicts changes in fractions of KillerOrange (yellow lines) or KillerRed-expressing (red lines) cells in the population. Cell fractions were normalized to the fractions in non-illuminated sample. Error bars represent SD, N = 3.

We also tested whether it is possible to use KillerOrange and KillerRed to independently ablate populations of mammalian cells. We mixed cells expressing the phototoxic protein (KillerRed or KillerOrange) with cells expressing EGFP, illuminated mixture with blue (447/20 nm) or orange (590/20 nm) light, cultivated cells for two days and then analyzed their fluorescence. As expected, we found a strong decrease in the fraction of KillerRed-expressing cells in the population that passed through the orange light irradiation (Fig 4). In contrast, KillerOrange-expressing cells were substantially removed from the population of cells that was illuminated with the blue light. Taken together, these results show that KillerOrange is phototoxic to mammalian cells and that KillerRed and KillerOrange can be used simultaneously to independently control fates of two cell populations.

## Discussion

Here we report the development of a new genetically encoded photosensitizer KillerOrange—an orange mutant of the red fluorescent protein KillerRed. KillerOrange is phototoxic upon blue and green light illumination and can be used simultaneously with KillerRed to independently control the fate of cell populations by tuning the light source wavelength.

KillerOrange possesses two spectral forms and absorbs light efficiently in the range from ~420 nm to ~530 nm. Based on the substantial ablation of KillerOrange-expressing cells in both 450–495 nm and 505–545 nm light-illuminated samples (Fig 3) we think that both spectral forms are phototoxic.

The blue-shifted spectra of KillerOrange makes it potentially more suitable for two-photon microscopy than the parental KillerRed. Also, the large Stokes shift of over 40 nm would make it possible to spectrally separate signals of KillerOrange from cyan and green fluorescent proteins when the proteins are being excited simultaneously by blue light. One can thus use green and cyan indicators to observe the effects of phototoxicity in real time without the need to change the excitation light.

With its broad absorption spectrum KillerOrange has a substantial overlap in absorbance with another genetically encoded photosensitizer—miniSOG. With both proteins being different in many aspects we believe that the choice between them should be based on particular experimental conditions. KillerOrange is brighter and possesses strongly red-shifted emission. Like all other fluorescent proteins, KillerOrange chromophore maturation does not depend on any cofactors except for molecular oxygen (which is in any case required for reactive oxygen species production). As for miniSOG, its phototoxicity is dependent on the presence of a cofactor, flavin mononucleotide, which abundance can vary between cell compartments and cell types and can depend on the physiological state of a cell. MiniSOG was successfully used for cell ablation in *C*. *elegans* [39] and for chromophore assisted light inactivation [40] but, for unknown reasons, was shown to be inefficient when applied to mouse tumor xenografts *in vivo* [41]. Also, miniSOG and KillerRed differ in mechanism of reactive species production. At the moment the mechanism of KillerOrange phototoxicity is unknown, but we believe it to be the Type I, similarly to the parental KillerRed. Thus, KillerOrange and miniSOG are possibly not equivalent, and should be chosen with regards to the experimental system.

We believe that the expansion of the palette of available genetically encoded photosensitizers will open up new possibilities. KillerOrange-KillerRed pair can potentially be used to independently ablate two cell populations during development, or to cut off two distinct neural circuits in the brain. This pair also promise the orthogonal optical control of the propagation of signaling cascades either by chromophore-assisted light inactivation of the participating proteins or by triggering cascades with hydrogen peroxide produced by KillerRed and likely KillerOrange.

Due to same-channel excitation compatibility with the majority of green fluorescent protein-based sensors KillerOrange can be used in systems where immediate registration of the toxic effects is required, such as nervous system or fast intracellular processes. Combination of various photosensitizers in one cassette, or KillerOrange-KillerRed tandem fusions, may enhance phototoxicity under white light irradiation and may be useful both as a research tool in biology and as a potential anti-tumor agent in photodynamic therapy.

## Supporting Information

S1 Fig Fluorescence decay of KillerOrange at various pH and room temperature. Fluorescence was excited at 467 nm and detected at 565 nm.
(EPS)

S2 Fig KillerOrange spectra. Absorption (**A**), fluorescence excitation (**C**) and emission (**B, D**) spectra of KillerOrange at various pH (pH values are indicated in the legend). Note the correspondence between the pH-dependent change of emission intensity (**B, D**) and the average fluorescence lifetimes (S1 Fig) measured at similar conditions.
(EPS)

S3 Fig Spectra of LEDs used for phototoxicity experiment. Absorption spectra of KillerOrange and KillerRed and emission spectra of LEDs used for phototoxicity experiments with KillerOrange, KillerRed and EGFP in bacteria. Blue-filled area represents normalized spectrum of “BDX” LED, green-filled—spectrum of “BGX” LED and orange-filled—spectrum of “GYX” LED.
(EPS)

S4 Fig Phototoxicity of KillerOrange and KillerRed at various light conditions. Illumination of mixtures KillerOrange, KillerRed and EGFP-expressing E.coli cells with 450–495 nm (**A**), 505–545 nm (**B**) and 540–600 nm (**C**) light. Error bars represent SD, N = 4.
(EPS)

S1 Text KillerOrange amino acid sequence. (DOCX)
