# Supplementary material for: KillerOrange, a Genetically Encoded Photosensitizer Activated by Blue and Green Light
Source: PLoS One. 2015 Dec 17;10(12):e0145287. doi: 10.1371/journal.pone.0145287 (PMC4683004; doi:10.1371/journal.pone.0145287)
Supplement: S1 Text — (DOCX) [file pone.0145287.s005.docx]

**S1 Text. KillerOrange amino acid sequence**

MECGPALFQSDMTFKIFIDGEVNGQKFTIVADGSSKFPHGDFNVHAVCETGKLPMSWKPICHLIQWGEPFFARYPDGISHFAQECFPEGLSIDRTVRFENDGTMTSHHTYELSDTCVVSRITVNCDGFQPDGPIMRDQLVDILPSETHMFPHGPNAVRQLAFIGFTTADGGLMMGHLDSKMTFNGSRAIEIPGPHFVTIITKQMRDTSDKRDHVCQREVAHAHSVPRITSAIGSDQD
